# Lithium-coated polymeric matrix as a minimum volume-change and dendrite-free lithium metal anode

**DOI:** 10.1038/ncomms10992

**Published:** 2016-03-18

**Authors:** Yayuan Liu, Dingchang Lin, Zheng Liang, Jie Zhao, Kai Yan, Yi Cui

**Affiliations:** 1 Department of Materials Science and Engineering, Stanford University, Stanford, California 94305, USA; 2 Stanford Institute for Materials and Energy Sciences, SLAC National Accelerator Laboratory, 2575 Sand Hill Road, Menlo Park, California 94025, USA

## Abstract

Lithium metal is the ideal anode for the next generation of high-energy-density batteries. Nevertheless, dendrite growth, side reactions and infinite relative volume change have prevented it from practical applications. Here, we demonstrate a promising metallic lithium anode design by infusing molten lithium into a polymeric matrix. The electrospun polyimide employed is stable against highly reactive molten lithium and, via a conformal layer of zinc oxide coating to render the surface lithiophilic, molten lithium can be drawn into the matrix, affording a nano-porous lithium electrode. Importantly, the polymeric backbone enables uniform lithium stripping/plating, which successfully confines lithium within the matrix, realizing minimum volume change and effective dendrite suppression. The porous electrode reduces the effective current density; thus, flat voltage profiles and stable cycling of more than 100 cycles is achieved even at a high current density of 5 mA cm^−2^ in both carbonate and ether electrolyte. The advantages of the porous, polymeric matrix provide important insights into the design principles of lithium metal anodes.

## Abstract (web summary)

Lithium metal is a promising anode for batteries, but problems such as volume change and dendrite growth currently prevent it from practical usage. Here, the authors overcome these problems by infusing molten lithium into a polymeric matrix via a lithiophilic coating to make a stable nanoporous lithium electrode.

## Graphical abstract

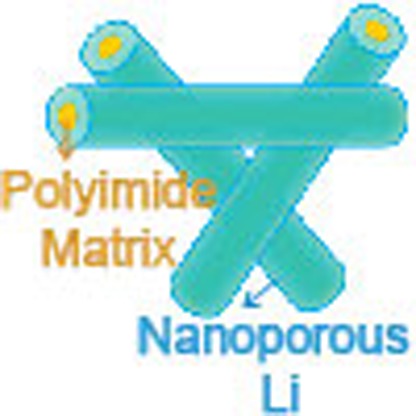

The ever-increasing demand for high-energy-density storage systems for transportation (electric vehicles), portable electronics and other applications has stimulated intensive research on rechargeable batteries that go beyond the conventional lithium (Li) ion chemistry^1^. Among all the possible options^2^,^3^, Li metal is the most ideal anode material due to its high theoretical capacity (3,860 mAh g^−1^) as well as its low electrochemical potential (−3.040 V versus standard hydrogen electrode)^4^,^5^. Despite the appealing properties, Li metal electrode has been plagued for decades with the problem of ramified growth during repeated stripping/plating and the associated electrolyte decomposition, which lead to serious safety concerns and poor battery cycling efficiency^6^,^7^.

It is well known that Li is highly reactive such that in liquid electrolyte it reacts spontaneously with solvent molecules and salt anions to form an insoluble layer of solid-electrolyte interphase (SEI)^8^. When SEI becomes stabilized to block electron transfer, this passivating film can slow down or, ideally, prevent the electrolyte from further decomposition. Nevertheless, as a ‘hostless' electrode, the Li metal anode has a virtually infinite relative volume change during stripping/plating, resulting in the mechanical instability of the SEI layer and the formation of cracks. The cracks expose fresh Li underneath and locally enhance the Li ion flux, leading to non-homogeneous Li growth (dendrite, filament, etc.) that can induce internal short circuit and thermal runaway with potential safety hazards^9^,^10^. Moreover, the large-surface-area, dendritic Li growth brings about a continuous loss of both working Li and electrolyte (recurrent SEI formation), which gives rise to low Coulombic efficiency (CE) and rapid capacity decay.

For the past four decades, continuous research on Li metal stripping/plating has deepened our understanding of the process, but has not helped in solving the above-mentioned problems in an effective manner^11^,^12^,^13^,^14^. On one hand, the use of solid electrolytes to suppress dendrite propagation remains premature in the current stage^15^,^16^,^17^, for they often fall short of meeting the high-power requirement at ambient temperature due to limited ionic conductivity^18^,^19^, together with issues such as large interfacial impedance^20^,^21^. On the other hand, the most common approaches to dendrite mitigation in liquid electrolyte focus on the stabilization of SEI via adjusting the electrolyte composition and additives^22^,^23^,^24^,^25^,^26^. Though proven to be effective, most additives will be continuously consumed during battery cycling so that the suppression effect is not fully sustainable^27^,^28^,^29^. Alternatively, the application of a mechanically stable artificial SEI coating such as polymer or solid-state blocking layers has been proposed^30^,^31^,^32^,^33^. For example, a promising nanoscale interfacial engineering approach has been demonstrated recently based on interconnected hollow carbon nanospheres, ultrathin two-dimensional boron nitride or oxidized polyacrylonitrile fibres to control the dendrite growth and improve the cycling CE^34^,^35^,^36^. Nevertheless, all these studies adopted the galvanostatic Li plating/stripping approach on a current collector, which is still unable to address the issue of infinite volume change since the electrodes expand during Li plating and shrink during stripping. In addition, contrary to Li-ion batteries where Li ions are stored in prelithiated cathodes, many of the intensively studied high-energy-density battery chemistries (e.g., Li-air and Li-S) involve cathodes in the non-lithiated form. Therefore, it is apparent that a metallic Li anode design with no volume change at the whole-electrode scale and long-term cycling stability in liquid electrolyte is of paramount research importance^3^.

Herein, we demonstrate a rational design of metallic Li anode that successfully achieves minimum volume change at the whole-electrode level and stable, dendrite-free Li cycling. Several important design principles are employed. Firstly, in order to realize negligible volume change, a chemically as well as electrochemically stable matrix is required to sustain a constant electrode volume during cycling. In addition, complete confinement of Li within the matrix is necessary to preserve a constant electrode dimension; therefore, the direct nucleation of Li on the top surface of the matrix should be prevented, as reported in previous studies^31^,^34^. Moreover, a porous electrode is desirable since a reduced local current density is beneficial to alleviate dendrite propagation^37^,^38^. Following the aforementioned rationale, a Li-coated polyimide (PI) matrix design for metallic Li anode is proposed. The electrospun polymeric fibres guarantee a chemically and electrochemically inert matrix, which is favourable to confine the stripping/plating of Li solely within the matrix. Notably, the choice of PI is rather unique for it is one of the only few high-performance polymers that exhibit excellent chemical stability, heat resistance and mechanical strength above the melting point of Li (180 °C)^39^. However, the wetting of molten Li on PI polymer is poor. By applying a layer of ZnO coating via atomic layer deposition (ALD) on the PI fibres, we discover that molten Li can react with ZnO and subsequently infuse into the PI matrix, resulting in a free-standing, current collector-free Li electrode. More importantly, we separate the conducting function from the matrix, where infused metallic Li itself serves as the only electron transport media. As a result, the electrically insulating surface after Li stripping effectively prevents the direct plating of Li on the top surface of the matrix in the subsequent cycle, bringing about a well-confined, dendrite-free Li stripping/plating behaviour that successfully addresses the problem of infinite volume change present in all the previous designs. Moreover, the obtained electrode is highly porous so that the reduced effective current density results in flat voltage profiles and stable cycling of at least 100 cycles in both carbonate- and ether-based electrolytes even at a high current density of 5 mA cm^−2^, which stands in stark contrast to the fluctuated and unstable cycling profile of bare Li foil electrodes.

## Results

### Fabrication of the Li-coated PI electrode

**Figure 1:**
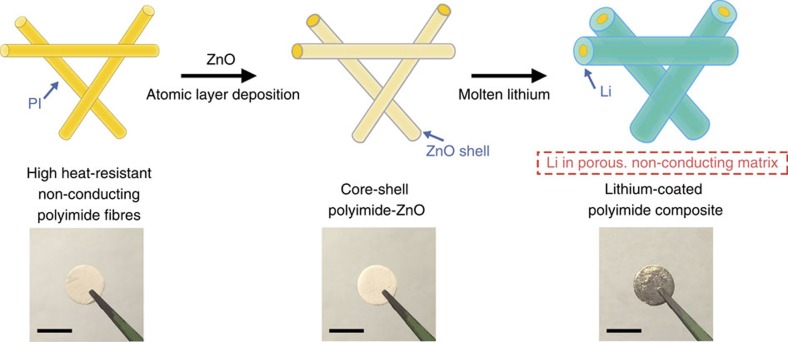
Schematic of the fabrication of the Li-coated PI matrix. Electrospun PI was coated with a layer of ZnO via ALD to form core-shell PI-ZnO. The existence of ZnO coating renders the matrix ‘lithiophilic' such that molten Li can steadily infuse into the matrix. The final structure of the electrode is Li coated onto a porous, non-conducting polymeric matrix. Scale bar, 1 cm.

Figure 1 illustrates the fabrication process of the Li-coated PI matrix electrode. We employed a facile electrospinning method to obtain the PI fibre matrix. Thermogravimetric analysis (Supplementary Fig. 1) confirmed that the electrospun PI fibre is stable up to 450 °C, which is well above the melting point of Li (180 °C). Such high heat resistance ensures that the matrix can withstand the temperature of molten Li in order to fabricate the metallic Li anode. Nevertheless, molten Li cannot wet the bare PI matrix (Supplementary Fig. 2). Due to the high surface tension of molten Li on PI fibre, a large driving force is needed for Li to infuse into the matrix. Rather than physical absorption, a surface chemical reaction that can afford much higher driving force is necessary. Through universal screening on materials that can undergo conversion reaction with Li, we found that a layer of conformal ZnO coating applied to the matrix via ALD can render the matrix wet by molten Li, or ‘lithiophilic'. Subsequently, when the core-shell PI-ZnO matrix was put into contact with molten Li, ZnO reacted with molten Li and, interestingly, extra Li can be drawn into the matrix, affording a Li-coated PI electrode.

### Characterization of the Li-coated PI electrode

**Figure 2:**
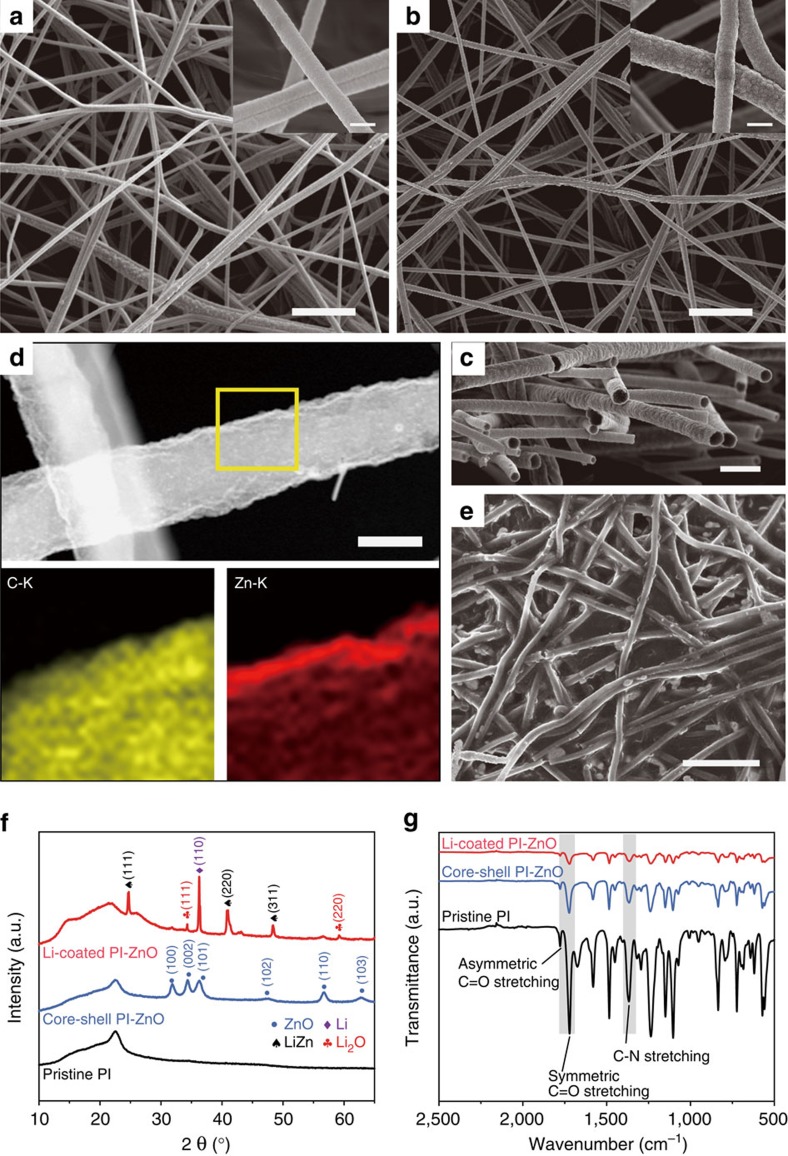
Characterization of the Li-coated PI electrode. SEM images of the electrospun PI fibres (**a**) before and (**b**) after ZnO coating. Scale bars, 5 μm; insets scale bars, 500 nm. (**c**) Cross-sectional SEM image of the core-shell PI-ZnO, where the conformal ZnO coating can be observed clearly from the contrast of the fibre cross-sections. Scale bar, 1 μm. (**d**) Scanning transmission electron microscopy (STEM) image of a single core-shell PI-ZnO fibre and the corresponding energy-dispersive X-ray (EDX) elemental mapping of C and Zn distribution. Scale bar, 200 nm. (**e**) SEM image of the Li-coated PI matrix, showing the porous nature of the Li electrode. Scale bar, 5 μm. (**f**) XRD spectra of the pristine PI, the core-shell PI-ZnO and the Li-coated PI-ZnO matrix, where the Li-coated PI-ZnO exhibited the signals of LiZn alloy, Li_2_O and metallic Li. (**g**) Fourier transform infrared spectra of the pristine PI, the core-shell PI-ZnO and the Li-coated PI-ZnO matrix (Li was scraped away to expose the underlying matrix in order to obtain the signal). The characteristic peaks of PI remained after ZnO and Li coating, indicating the stability of the polymeric matrix.

The morphology of the electrospun PI fibres was characterized using scanning electron microscopy (SEM). As can be seen from Fig. 2a, the fibres were continuous and uniform in general with a diameter of ∼400 nm. After ALD coating, the surface of the fibres roughened due to the accumulation of a conformal layer of ZnO nanoparticles (Fig. 2b). Evident PI-ZnO core-shell structure can be observed from the cross-sectional SEM image (Fig. 2c), where the PI core appeared darker in colour and the thickness of the ZnO shell was measured to be ∼30 nm (Supplementary Fig. 3). Scanning transmission electron microscopy energy-dispersive X-ray elemental mapping (Fig. 2d) as well as line scan (Supplementary Fig. 4) resolved the distribution of C (from the PI backbone) and Zn (from the ZnO coating), further confirming the core-shell structure of the fibre matrix after ALD. Figure 2e shows the SEM top view of the PI matrix after Li coating. It appears that Li was drawn into the matrix preferentially along the fibres, and hence the matrix was not densely coated with Li. The porous nature of the resulting matrix can be further revealed from the cross-sectional SEM image (Supplementary Fig. 5), where obvious pores can be observed. X-ray diffraction (XRD) was employed to understand the compositional evolution of the matrix (Fig. 2f). As can be seen clearly from the XRD spectra, the ZnO layer reacted with molten Li to form LiZn alloy and Li_2_O during the Li coating process^40^. Since the electron percolation pathway of the alloy particles within the non-conducting Li_2_O matrix is generally limited it is justified to believe that the matrix remained low in electrical conductivity compared to metallic Li after Li coating^41^,^42^. Such a porous and electrically insulating matrix renders favourable electrochemical features to the resulting Li electrode, which will be elaborated in detail in later sections.

It is important to ensure the stability of the polymeric backbone in contact with the highly reductive molten Li. Therefore, Fourier transform infrared spectroscopy was employed (Fig. 2g), where the existence of PI can be identified from three characteristic peaks corresponding to asymmetric C=O stretching, symmetric C=O stretching and C-N stretching^43^. After ALD and the subsequent contact with molten Li, the transmittance intensity was reduced due to the coating layers, while the three characteristic peaks remained, confirming the intact polymeric matrix. In addition, neither anodic nor cathodic decomposition peaks can be observed from the cyclic voltammetry of the pristine PI (Supplementary Fig. 6), indicating the stability of the polymeric matrix towards electrochemical cycling.

The capacity of the Li electrode was determined to be above 2,000 mAh g^−1^ based on the weight of the whole composite electrode via Li stripping (Supplementary Fig. 7). Thus, the existence of the matrix did not seriously compromise the high specific capacity of the Li anode. Noticeably, by adjusting the thickness of the electrospun matrix, which can be done easily by changing the electrospinning time, the thickness of the final Li electrode can be tuned accordingly with great ease to match the capacity of the battery cathode (Supplementary Fig. 8), manifesting the facileness of our proposed method for real applications.

### Well-confined Li stripping/plating within the matrix

**Figure 3:**
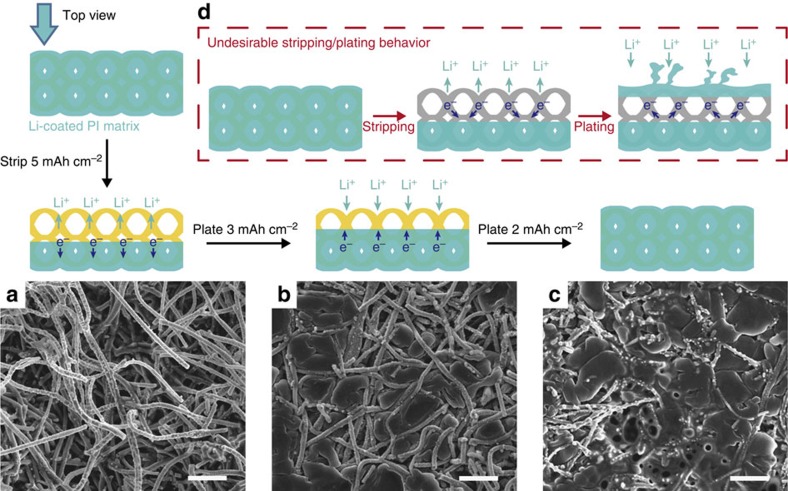
Well-confined stripping/plating behaviour of the Li-coated PI matrix. Top-view SEM images of (**a**) the exposed top fibres of the Li-coated PI electrode after stripping away 5 mAh cm^−2^ Li; (**b**) exposed top fibres partially filled with Li when plating 3 mAh cm^−2^ Li back and (**c**) completely filled PI matrix after plating an additional 2 mAh cm^−2^ Li back (current density 1 mA cm^−2^, in EC/DEC). The polymeric matrix ensures that Li is dissolved and deposited from the underlying conductive Li substrate and, as a result, Li is effectively confined into the matrix. (**d**) Schematic illustrating the alternative undesirable Li stripping/plating behaviour where, after stripping, Li nucleate on the top surface, leading to volume change and dendrites shooting out of the matrix. Scale bars, 5 μm.

We investigated the stripping/plating process of the Li-coated PI matrix using a two-electrode symmetric cell configuration assembled in 2,032 coin cells with carbonate-based electrolyte (1 M lithium hexafluorophosphate (LiPF_6_) in 1:1 ethylene carbonate (EC)/diethyl carbonate (DEC), BASF). Interestingly, the electrode exhibited a well-confined stripping/plating behaviour (Fig. 3). Top fibres of the matrix were exposed after stripping away 5 mAh cm^−2^ Li at a current density of 1 mA cm^−2^ (Fig. 3a), which indicates that the top Li layers were dissolved more favourably during stripping. Subsequently, when 3 mAh cm^−2^ Li was plated, Li was observed to be deposited into the matrix and partially fill the space between the fibres (Fig. 3b). Finally, when all the stripped Li was plated back (Fig. 3c), the top surface of the matrix was covered again by Li (similar to Fig. 2e) with no discernable dendrites. The well-confined plating behaviour can be rationalized by the removal of the conductive Li component in the prior stripping process, and thus the exposure of the electrically insulating PI surface. Since Li plating only occurs where electrons meet Li ions, the exposed insulating surface was rendered unfavourable for Li nucleation. Instead, the metallic Li confined within the matrix served as the only electron conductor such that the deposition of Li occurred majorly on the underlying reserved Li. In addition, the much larger effective surface area (Supplementary Fig. 9) lowered the overall deposition barrier, thus preventing the formation of ‘hot spot'. As a result, uneven Li deposition can be suppressed. On the contrary, if the electrons could be efficiently transported to the electrolyte-facing top surface or the electrodes exhibited limited surface area, undesirable Li stripping/plating behaviour may occur after recurrent cycles (Fig. 3d), as discussed in previous studies^31^,^34^. Direct Li nucleation on the top surface might be easier due to the high availability of both electrons and Li ions, which provides favourable sites for dendrite growth while leaving the interior voids empty.

### Dendrite-free cycling with minimum volume change

**Figure 4:**
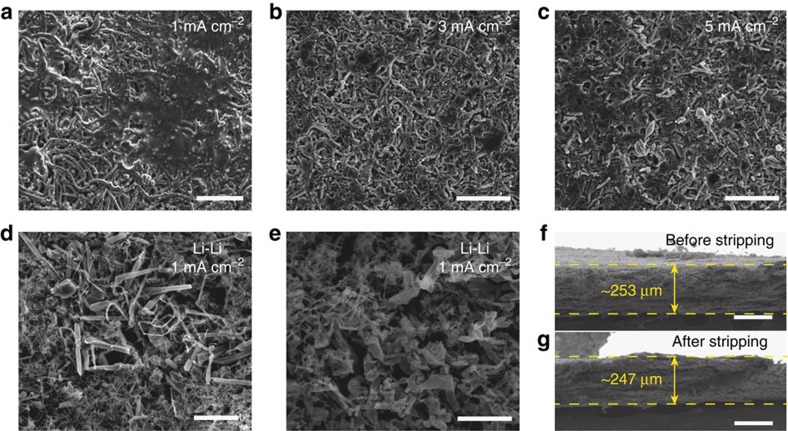
Morphology of the Li electrodes after cycling at different current densities. Top-view SEM images of the Li-coated PI matrix after 10 cycles of stripping/plating in EC/DEC at a current density of (**a**) 1 mA cm^−2^, (**b**) 3 mA cm^−2^ and (**c**) 5 mA cm^−2^. (**d**,**e**) Top-view SEM images of the bare Li electrode after 10 cycles of stripping/plating in EC/DEC at a current density of 1 mA cm^−2^ with large amounts of mossy Li dendrites. Cross-sectional SEM images of the Li-coated PI matrix (**f**) before and (**g**) after complete Li stripping, from which no significant volume change can be seen. (Note that the uniform fibrous features in **a**–**c** are not dendrites but the fibrous matrix, which are distinctly different from the non-uniform, random-sized mossy Li dendrites in **d**,**e**). Scale bars, (**a**–**d**) 10 μm, (**e**) 5 μm, (**f**,**g**) 200 μm.

The morphology of the top surface of the Li-coated PI matrix was studied after 10 cycles of galvanostatic stripping/plating in EC/DEC (Fig. 4a–c, Supplementary Fig. 10). Due to the above-mentioned well-confined Li cycling behaviour, the surface of the Li-coated PI electrode remained consistently flat even at a high current density of 5 mA cm^−2^ (note that the uniform fibrous features in Fig. 4a–c are the fibre matrix and shall not be mistaken as Li dendrites). Moreover, no excessive dendrite formation can be observed after long-term cycling of 100 cycles (Supplementary Fig. 11). On the contrary, for the bare Li electrodes, rough surface and excessive mossy Li growth can be observed after 10 cycles even at a relatively low current density of 1 mA cm^−2^. Such drastically different result further demonstrates the merit of the PI matrix on dendrite suppression.

Moreover, due to the existence of the host matrix, the issue of infinite volume change associated with the ‘hostless' Li stripping/plating can now be solved. Even with the complete stripping of Li, the change in electrode thickness was minimal. For example, as shown in Fig. 4f,g, the electrode size was on average ∼253 μm before stripping and remained at ∼247 μm after complete stripping, which was merely ∼2.4% of change (a relatively thick electrode was chosen for the more precise determination of thickness variation). However, for bare Li foil, 1 mAh cm^−2^ capacity represents ∼4.85 μm thickness of Li (see Supplementary Method). Therefore, at least tens of microns of electrode thickness fluctuation can be expected for merely a single layer of Li electrode in a commercial cell. Considering the conventionally applied stacking or rolling battery configuration with multiple layers, the accumulated dimension fluctuation can be tremendous. It is noted that for later cycles, due to the formation of dendritic Li and thus a porous electrode, the dimension fluctuation can be even larger. Thus, it is apparent that the existence of a stable matrix and the well-confined Li cycling behaviour are essential to alleviate the electrode-level volume change, addressing the potential safety concerns.

### Electrochemical cycling stability

**Figure 5:**
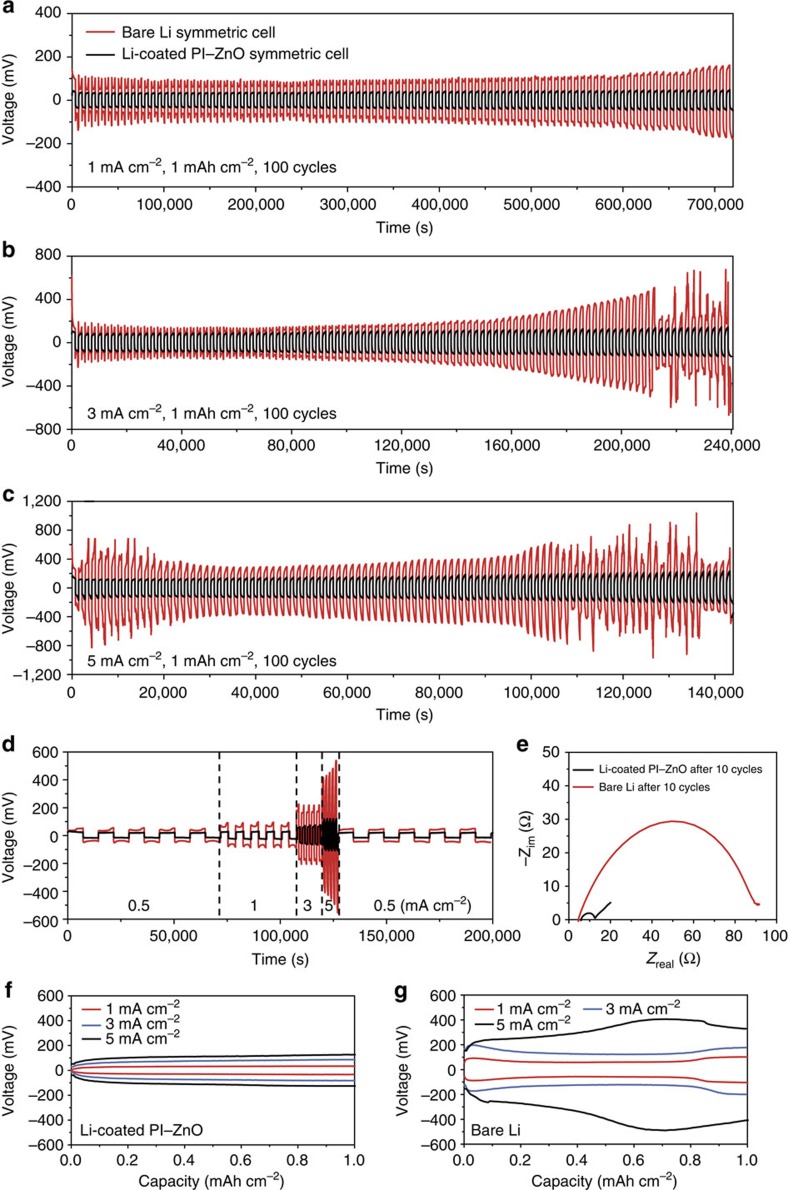
Electrochemical characterization in EC/DEC electrolyte. Comparison of the cycling stability of the Li-coated PI matrix and the bare Li electrode at a current density of (**a**) 1 mA cm^−2^, (**b**) 3 mA cm^−2^ and (**c**) 5 mA cm^−2^. (**d**) Rate performance of the Li-coated PI matrix and the bare Li electrode. (**e**) Nyquist plot of the impedance spectra of the symmetrical Li-coated PI matrix and the bare Li cell after 10 cycles at a current density of 1 mA cm^−2^. Voltage profiles of (**f**) Li-coated PI matrix and (**g**) bare Li electrode at different current densities after 10 cycles. The amount of Li cycled was 1 mAh cm^−2^ in all cases.

The galvanostatic cycling performance of the Li-coated PI matrix was studied in both carbonate (EC/DEC) and ether (1 M lithium bis(trifluoromethanesulfonyl)imide in 1:1 w/w 1,3-dioxolane (DOL)/1,2-dimethoxyethane (DME) with 1 wt% lithium nitrate) based electrolyte and compared with bare Li electrode (Fig. 5, Supplementary Figs 12–18). At a current density of 1 mA cm^−2^ in EC/DEC (Fig. 5a), the symmetrical cell of bare Li exhibited a large Li stripping/plating overpotential (>100 mV versus Li^+^/Li), which increased considerably within the first 100 cycles (>170 mV in the 100th cycle). In contrast, the Li-coated PI matrix not only showed a much lower overpotential (∼35 mV in the initial cycle) but also achieved very stable cycling for at least 100 cycles (∼40 mV overpotential in the 100th cycle). The difference in cycling stability (Fig. 5b,c) and overpotential (Fig. 5d) became increasingly pronounced at higher current densities. The stripping/plating overpotential for Li-coated PI matrix was ∼70 and ∼110 mV at a current densities of 3 and 5 mA cm^−2^, respectively, and the values remained constant within 100 cycles. However, the bare Li electrode succumbed to substantial voltage fluctuation at only 88 cycles at a current density of 3 mA cm^−2^ and 75 cycles at a current density of 5 mA cm^−2^, which might be attributed to possible dendrite-induced soft short circuit. Greatly improved cycling stability was also observed as we increased the cycling capacity to 3 mAh cm^−2^ (Supplementary Fig. 12). Similarly, in DOL/DME electrolyte, the Li-coated PI matrix again outperformed the bare Li electrode (Supplementary Fig. 13). Noticeably, although it is generally recognized that DOL can improve the cycling life of Li metal anodes due to the formation of a relatively flexible oligomer SEI^44^, the bare Li electrode still exhibited a necking behaviour (the overpotential first decreases, then increases) during cycling, which is a characteristic sign for dendrite formation in early stage and SEI accumulation later^37^. Nevertheless, the Li-coated PI matrix maintained flat, constant cycling profiles and reduced overpotential at all current densities. Such exceptional long-term cycling performance is a good indicator of the superior CE and more uniform Li deposition/dissolution of the Li-coated PI electrode. Finally, since it is technically challenging to directly determine the CE of electrodes with pre-stored Li through Li plating/stripping as in previous studies^27^,^34^, we paired our electrode with high-areal-capacity Li_4_Ti_5_O_12_ (LTO, ∼3 mAh cm^−2^) electrode as an indirect method to study the CE of the electrode (Supplementary Fig. 14). Li-coated PI electrode (∼10 mAh cm^−2^) exhibited quite stable cycling comparable to the highly oversized Li metal counterpart (750 μm Li), while bare 50 μm Li foil (∼10 mAh cm^−2^) started to decay at ∼20 cycles. The results gave rise to semiquantitative CE values and illustrate the superior CE of the Li-coated PI electrode (see Supplementary Note 1).

## Discussion

Two key factors contributed to the excellent electrochemical performance of the Li-coated PI matrix, namely, the porous nature of the electrode and the non-conducting nature of the exposed matrix surface after Li stripping. As mentioned previously, molten Li was drawn into the matrix preferentially along the fibres during the Li coating step, resulting in a porous Li electrode. The high porosity can be further confirmed by the fast electrolyte uptake during cell assembly (Supplementary Fig. 15). Such high porosity increased the surface area of the electrode, which can in turn significantly reduce the effective current density during cycling. As a result, the Li stripping/plating overpotential was much smaller for the Li-coated PI matrix, especially at high current densities (Fig. 5d). Correspondingly, the electrochemical impedance spectroscopy revealed a much reduced interfacial charge transfer resistance for the Li-coated PI matrix compared to bare Li (∼10 times lower in EC/DEC, Fig. 5e and Supplementary Fig. 16). More importantly, the reduced effective current density rendered the Li-coated PI matrix a flat stripping/plating voltage profile (Fig. 5f,g and Supplementary Fig. 17). For the bare Li electrode, large ‘overpotential bumps' at the beginning and the end of each stripping or plating process can be observed, especially for the early cycles (Supplementary Fig. 18). This phenomenon can be explained by the high specific kinetic hindrance for non-uniform Li dissolution/deposition at high current density, which has been investigated in great detail in previous reports^37^,^45^. The fluctuation attenuated in later cycles due to the formation of mossy Li, which increased the surface area of the electrode, reducing the effective current density. However, when compared to the fluctuated profile of the bare Li electrode, the voltage profile of Li-coated PI was flat at all current densities throughout the cycling, clearly demonstrating the advantages of the porous electrode. In addition, it is well known that the effective current density during Li stripping/plating has a crucial impact on the dendrite formation and growth^37^,^46^. Lower effective current density results in reduced electrolyte decomposition and related SEI formation during cycling so as to suppress the dendrite growth^37^. Therefore, the porous electrode structure, in addition to the non-conducting polymeric matrix, which led to the well-confined Li dissolution/deposition behaviour, effectively ensured the dendrite-free cycling of the Li-coated PI electrode, giving rise to stable long-term performance.

In conclusion, our work demonstrates the method of obtaining a free-standing, porous metallic Li anode by infusing molten Li into a core-shell PI-ZnO matrix. The excellent heat resistance and chemical stability of the PI fibres guaranteed the structural integrity of the matrix during Li coating and the subsequent battery cycling, while the conformal ALD ZnO coating provided the driving force for the molten Li infusion. Noticeably, the exposed non-conducting fibres after Li stripping prevented the direct plating of Li on the top surface of the electrode, which effectively confined Li within the matrix. In this manner, dendrite-free and minimum-volume-change Li stripping/plating can be successfully achieved, addressing the biggest concerns regarding Li metal anode. Remarkably, different from the dense bare Li electrode, the large porosity of the Li-coated PI electrode considerably decreased the effective current density during cycling. As a result, flat voltage profiles and long-term cycling stability can be realized even at a high current density of 5 mA cm^−2^. The benefits of a non-conducting polymeric matrix and electrode porosity shed new light on the design principles of metallic Li anodes and open up new opportunities to the realization of the next-generation high-energy-density battery systems based on Li metal chemistries.

## Methods

### Electrospinning of the PI matrix

15 wt% PI powder (DuPont CP-0650) was dissolved in *N*-methyl-2-pyrrolidone and stirred at 750 r.p.m. in a 60 °C oil bath overnight to afford a homogeneous solution. Subsequently, the solution was loaded into a glass syringe with a stainless steel needle tip, which is connected to a voltage supply (ES30P-5 W, Gamma High Voltage Research). The applied potential on the needle was 15 kV, the distance between the needle tip and the graphite paper collector was 15 cm and the pumping rate was 10 μl min^−1^. In addition, a negative voltage of 1 kV was applied at the collector to improve the homogeneity of the electrospun film. The thickness of the electrospun PI matrix can be tuned easily by adjusting the electrospinning time.

### Atomic layer deposition

The conformal ZnO layer was coated on the PI fibres via ALD using the Cambridge Nanotech Savannah S100 at 80 °C with diethyl zinc (DEZn) and DI water as precursors. The pulse times for DEZn and DI water were 15 ms each, with 60 s waiting between each pulse. Approximately 300 cycles were needed to obtain a ZnO coating with desirable Li wetting property.

### Li coating of the core-shell PI-ZnO matrix

The Li coating process was carried out in an argon-filled glovebox with sub-p.p.m. O_2_ level. In a typical process, freshly scraped Li foil (99.9%, Alfa Aesar) was put into a stainless-steel crucible and heated to melt on a hotplate (VWR). Subsequently, the edge of the core-shell PI-ZnO matrix (punched into 1-cm^2^ discs) was put into contact with the molten Li. Li can steadily climb up and wet the whole matrix, affording the final Li electrode.

### Electrochemical measurements

The processes of Li stripping and plating were studied using a symmetric cell configuration by assembling the electrodes into 2,032-type coin cells. The electrolytes employed were either 1 M lithium hexafluorophosphate (LiPF_6_) in 1:1 EC/DEC (BASF Selectilyte LP40) or 1 M lithium bis(trifluoromethanesulfonyl)imide in 1:1 w/w DOL/DME with 1 wt% lithium nitrate additive. The separator used was Celgard 2325 (25 μm PP/PE/PP). The control bare Li cells were assembled using freshly scraped Li foil. Galvanostatic cycling was conducted on a standard eight-channel battery tester (Wuhan LAND Electronics Co., Ltd.). A constant current was applied to the electrodes during repeated stripping/plating while the potential was recorded over time. The impedance measurements were carried out using a Biologic VMP3 multichannel system. For the half-cell study, the LTO electrodes were prepared by mixing LTO (MTI), polyvinylidene fluoride (MTI) and carbon black (TIMCAL) in the ratio of 8:1:1 with *N*-methyl-2-pyrrolidone as the solvent. The areal mass loading of the LTO electrodes was∼20 mg cm^−2^.

### Characterization

SEM images were taken using a FEI XL30 Sirion scanning electron microscope at an acceleration voltage of 5 kV. In order to observe the surface morphology of Li after cycling, the electrodes were disassembled from the coil cell in the glovebox followed by gentle rinse in DOL. Scanning transmission electron microscopy images, the corresponding energy-dispersive X-ray elemental mapping and line scan were obtained on a FEI Tecnai G2 F20 X-TWIN. XRD patterns were recorded on a PANalytical X'Pert instrument. Noticeably, the Li electrode was loaded on a glass slide and covered with Kapton tape during XRD measurements to avoid direct contact with air. Fourier transform infrared spectra were recorded on a Nicolet iS50 FT/IR Spectrometer (Thermo Scientific). Thermogravimetric analysis was performed on a TA Instrument Q500 TGA in air at a heating rate of 5 °C min^−1^. N_2_ sorption studies were performed using a Micromeritics ASAP 2,020 adsorption apparatus at 77 K and at pressure up to 1 bar after the samples were first degassed at 180 °C overnight. The Brunauer-Emmett-Teller surface area was calculated using the adsorption data in a relative pressure ranging from 0.1 to 0.3.

## Additional information

**How to cite this article:** Liu, Y. *et al*. Lithium-coated polymeric matrix as a minimum volume-change and dendrite-free lithium metal anode. *Nat. Commun.* 7:10992 doi: 10.1038/ncomms10992 (2016).

## Supplementary Material

Supplementary Information Supplementary Figures 1-18, Supplementary Note 1, Supplementary Methods and Supplementary References.
