# Supplementary material for: Lithium-coated polymeric matrix as a minimum volume-change and dendrite-free lithium metal anode
Source: Nat Commun. 2016 Mar 18;7:10992. doi: 10.1038/ncomms10992 (PMC4802050; doi:10.1038/ncomms10992)
Supplement: Supplementary Information — Supplementary Figures 1-18, Supplementary Note 1, Supplementary Methods and Supplementary References. [file ncomms10992-s1.pdf]

Supplementary Figures

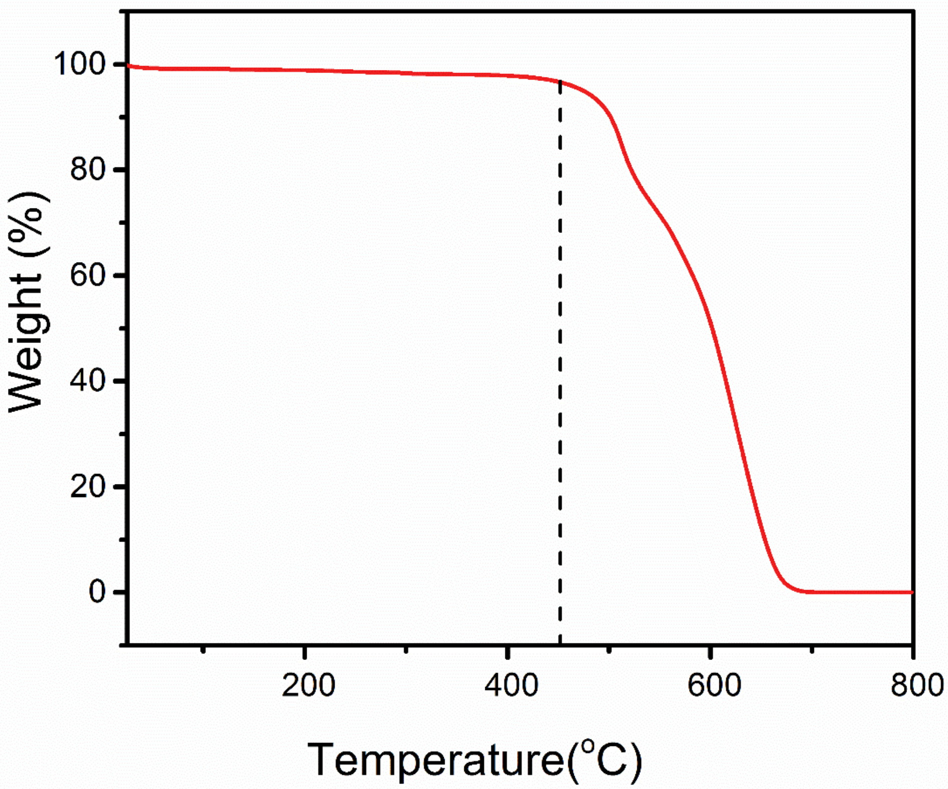

Supplementary Figure 1. TGA curve of the electrospun PI matrix in air atmosphere.

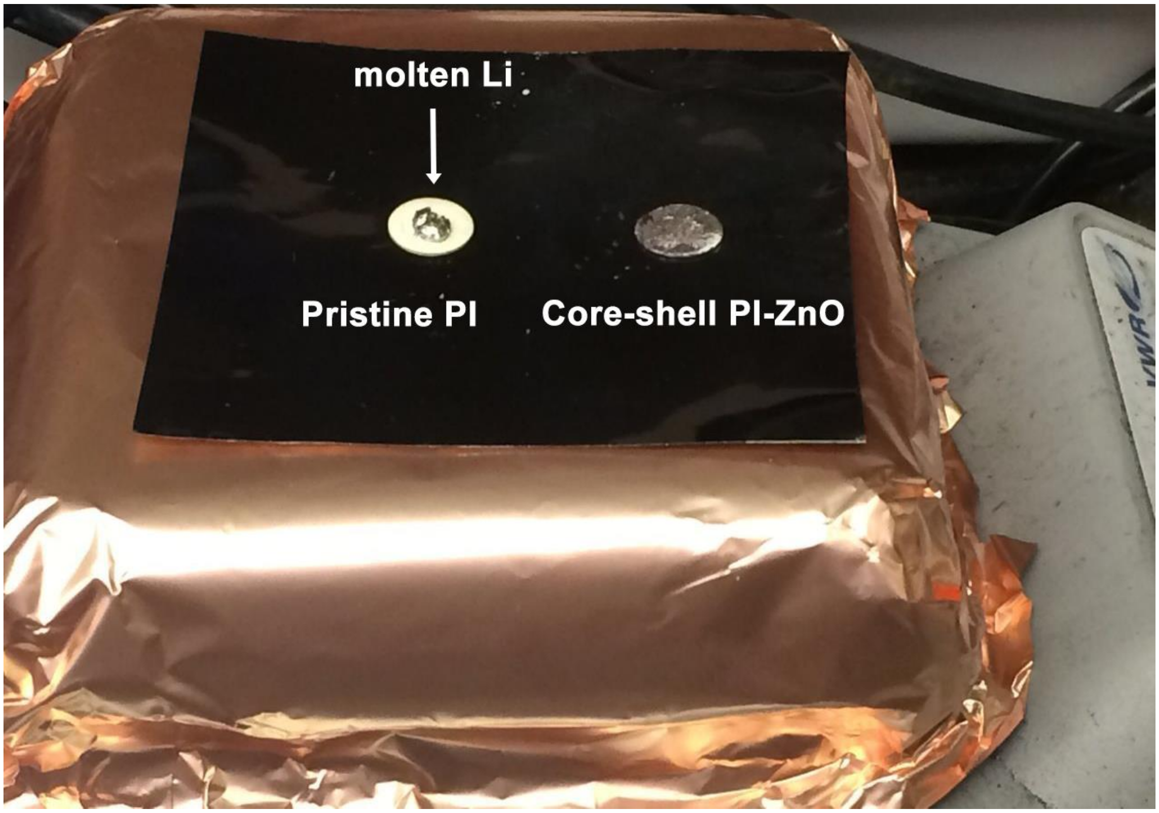

Supplementary Figure 2. Photo of a bare PI matrix and a core-shell PI-ZnO matrix in molten Li indicating the difference in Li wettability.

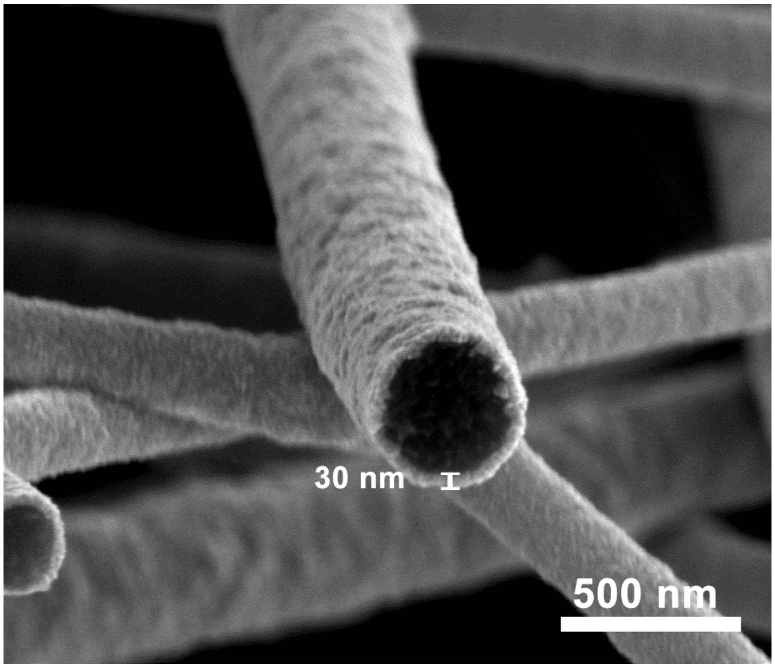

Supplementary Figure 3. Cross-sectional SEM image of the core-shell PI-ZnO fiber.

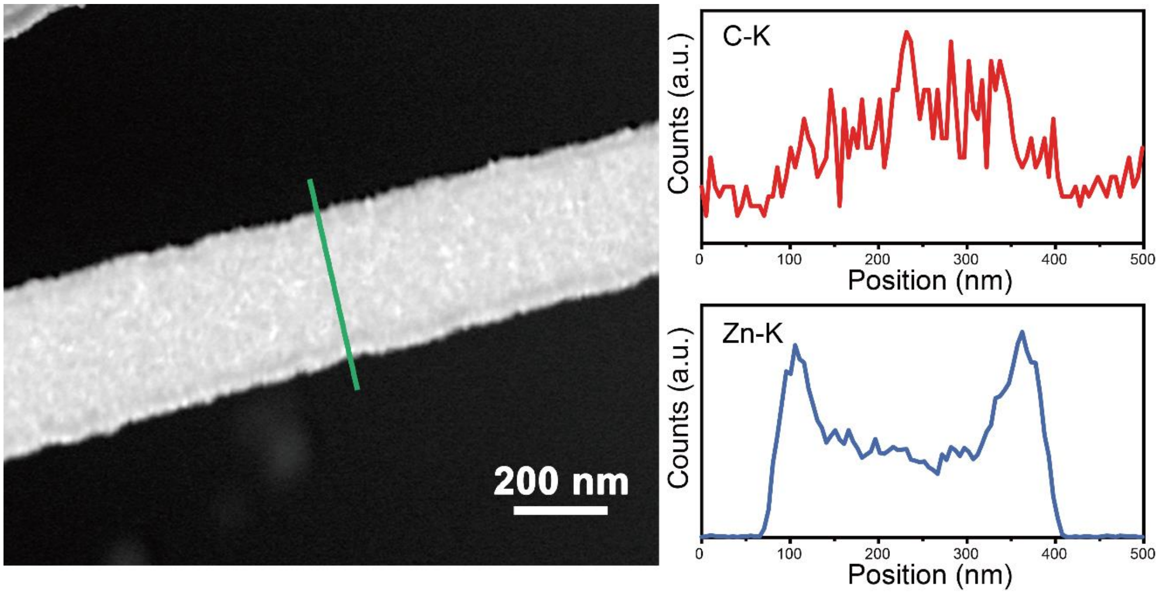

Supplementary Figure 4. EDX line scan of the PI fiber after ZnO ALD resolving the core-shell structure of the fiber. (C signal was originated from the PI polymeric core and the Zn signal was originated from the ZnO shell).

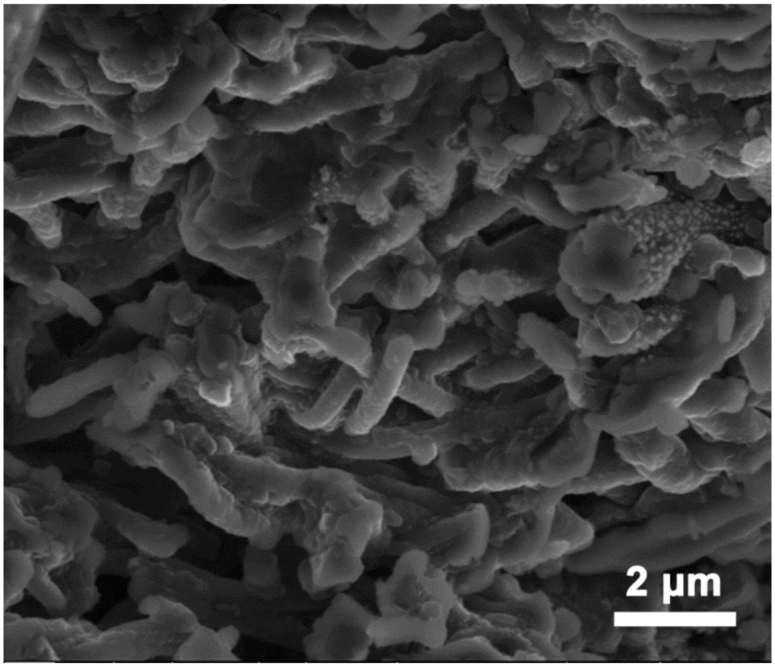

Supplementary Figure 5. Cross-sectional SEM image of the Li-coated PI matrix.

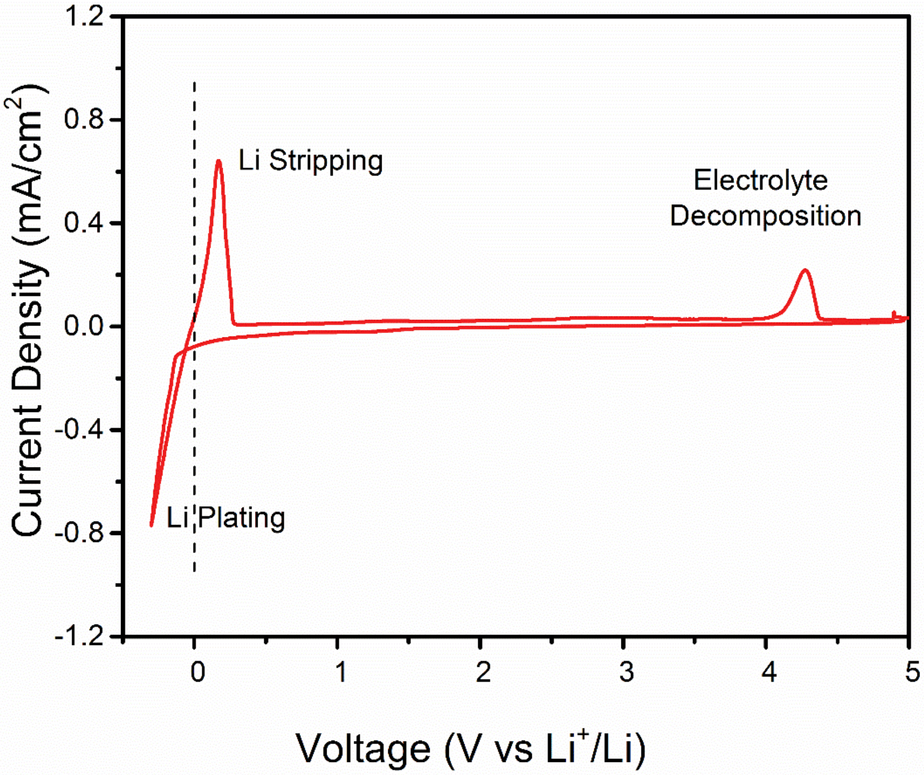

Supplementary Figure 6. CV scan of pristine PI at a scan rate of 1 mV s^{-1}.

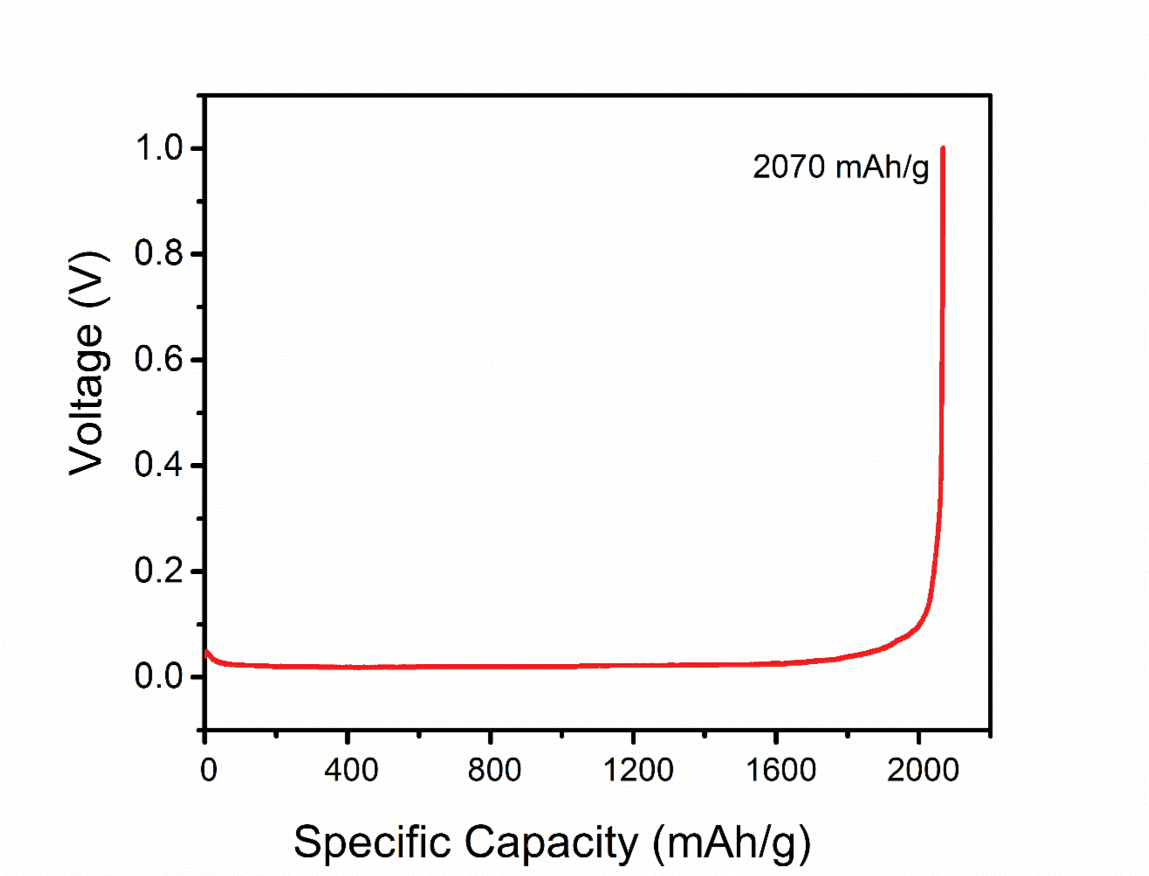

Supplementary Figure 7. A typical Li stripping curve of the Li-coated PI matrix with a stripping current density of 0.25 mA cm^{-2}.

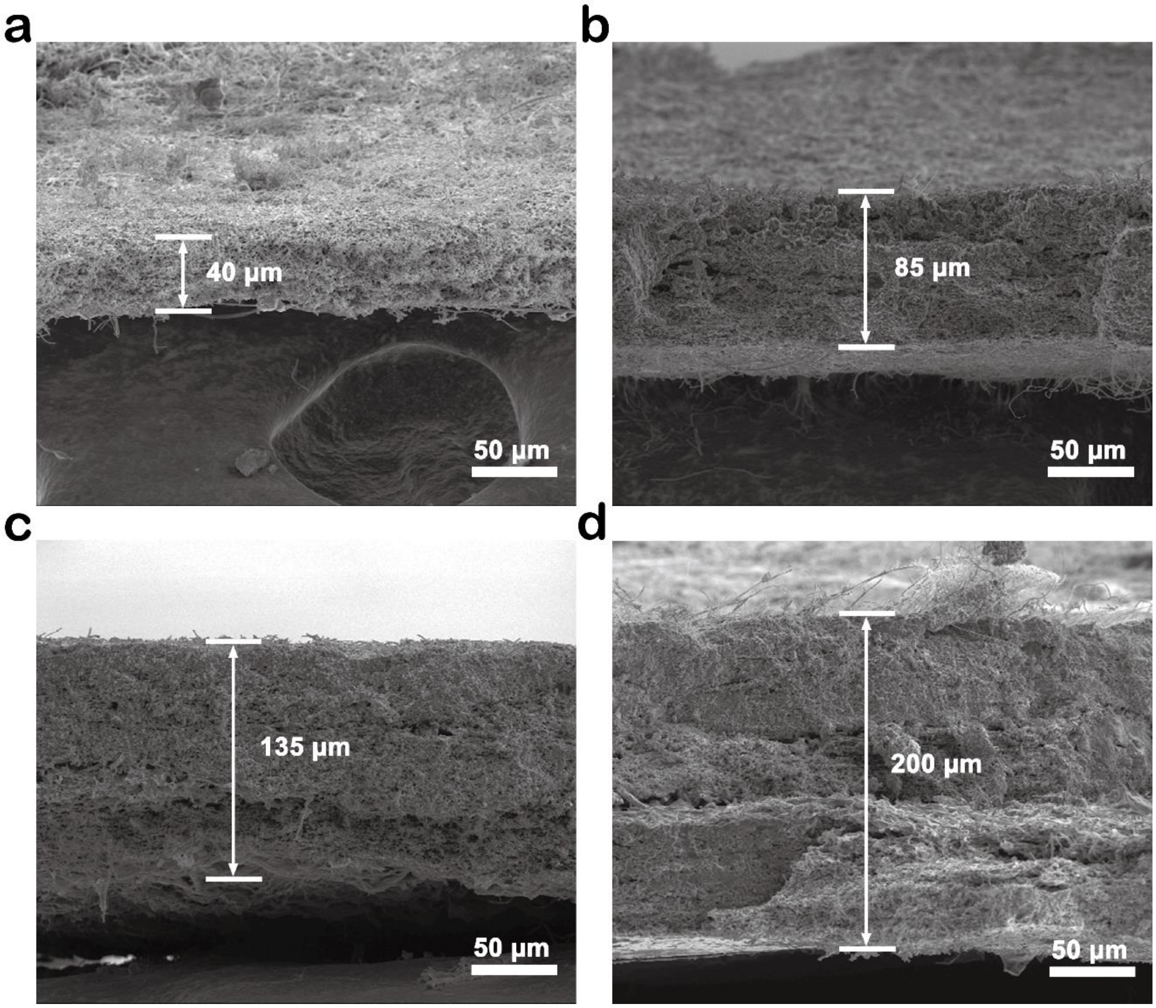

Supplementary Figure 8. Cross-sectional SEM images of the Li-coated PI matrix with different thicknesses.

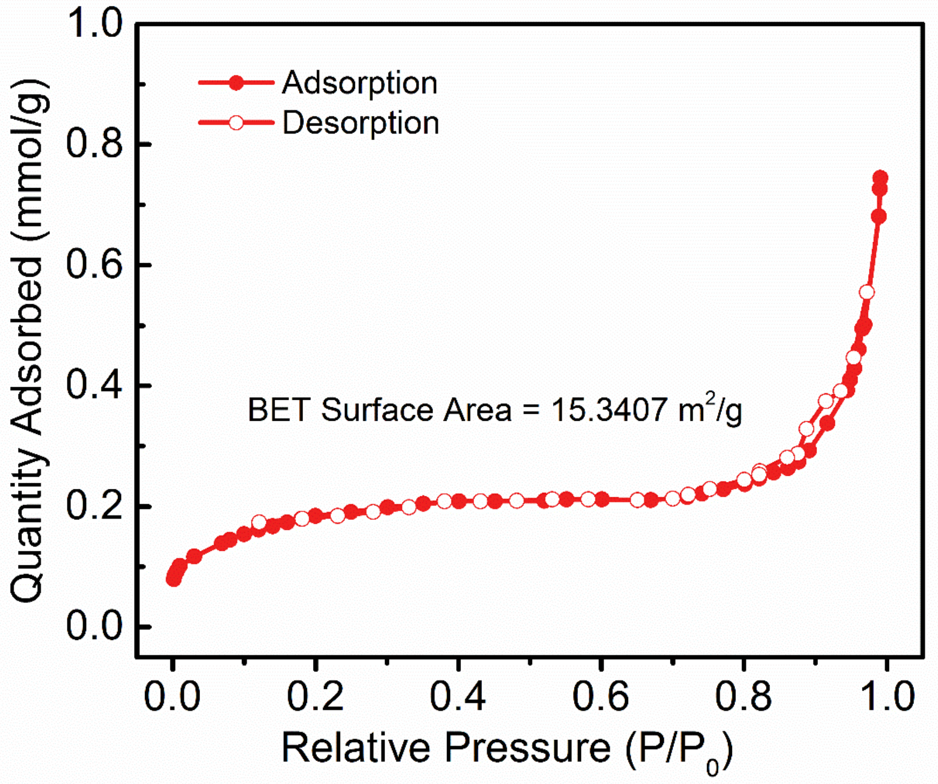

Supplementary Figure 9. N_2 adsorption-desorption isotherm of ZnO coated PI matrix.

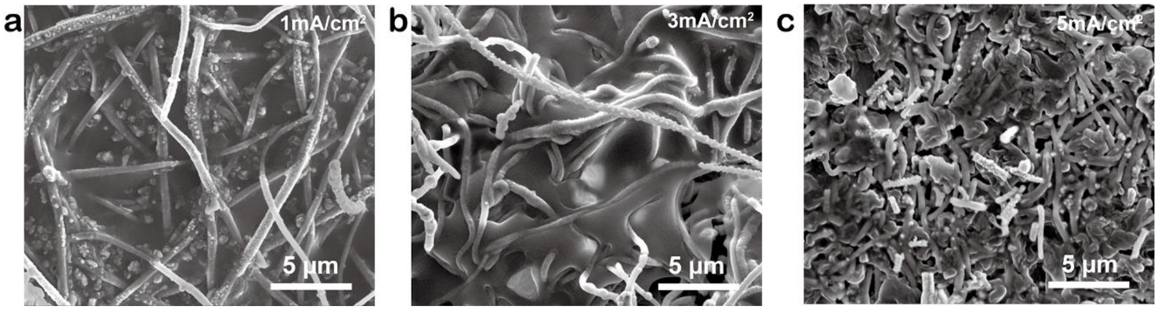

Supplementary Figure 10. High magnification top view SEM images of the Li-coated PI matrix after 10 cycles of stripping/plating in EC/DEC at a current density of (a) 1 mA cm^{-2}, (b) 3 mA cm^{-2} and (c) 5 mA cm^{-2} with a capacity of 1 mAh cm^{-2}.

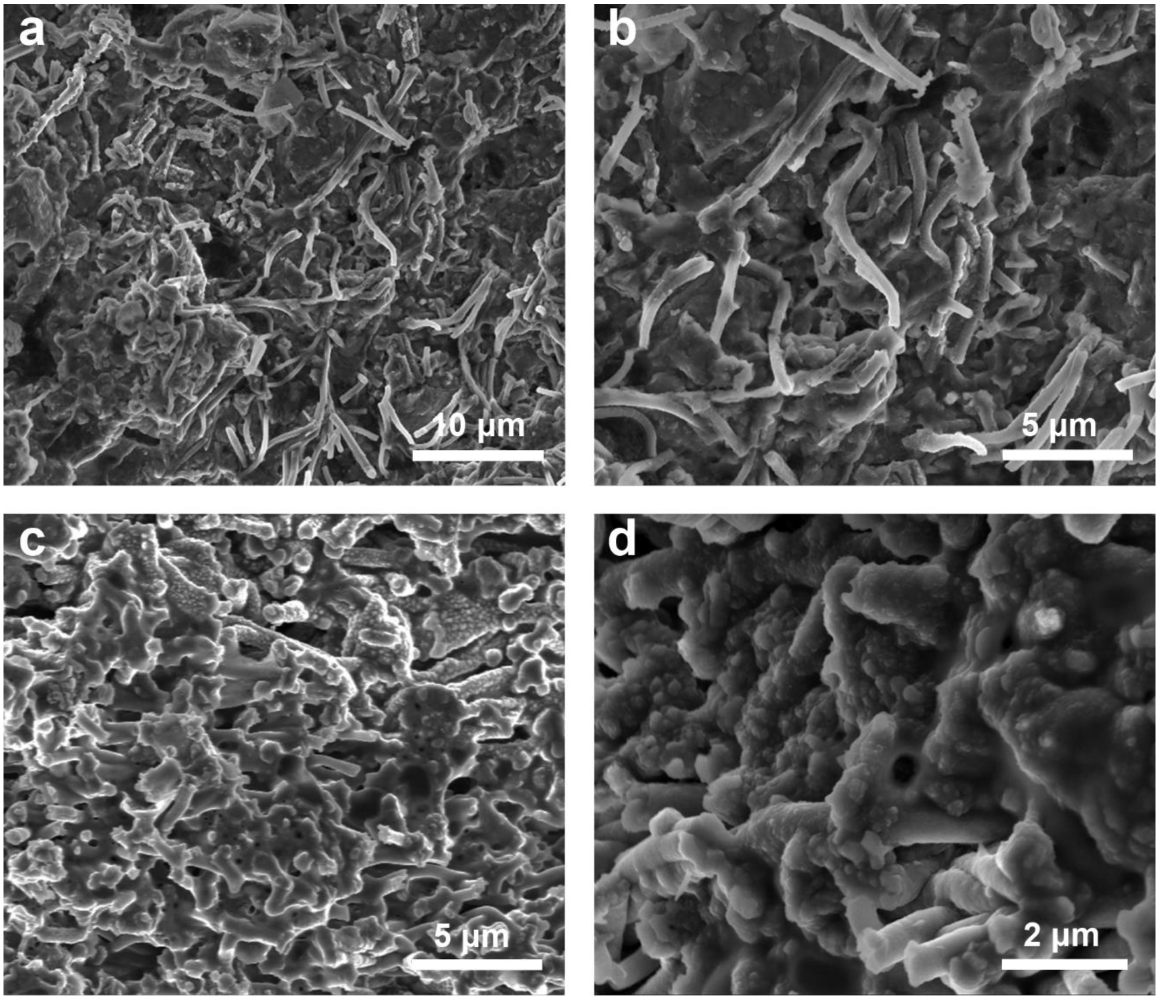

Supplementary Figure 11. SEM (a, b) top view and (c, d) cross-sectional view images of the Li-coated PI electrode after 100 galvanostatic stripping/plating cycles in EC/DEC at a current density of 1 mA cm^{-2} and a capacity of 1 mAh cm^{-2}.

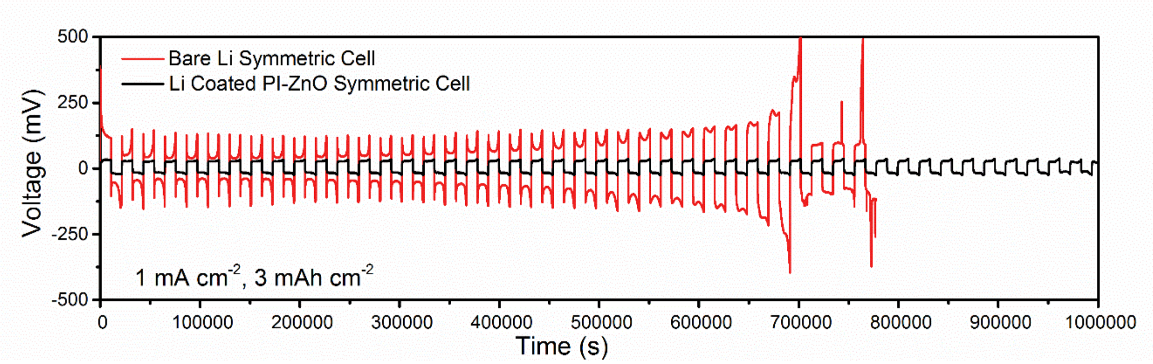

Supplementary Figure 12. Comparison of the cycling stability of the Li-coated PI matrix and the bare Li electrode at a current density of 1 mA cm^{-2} in EC/DEC electrolyte. The amount of Li cycled was 3 mAh cm^{-2}.

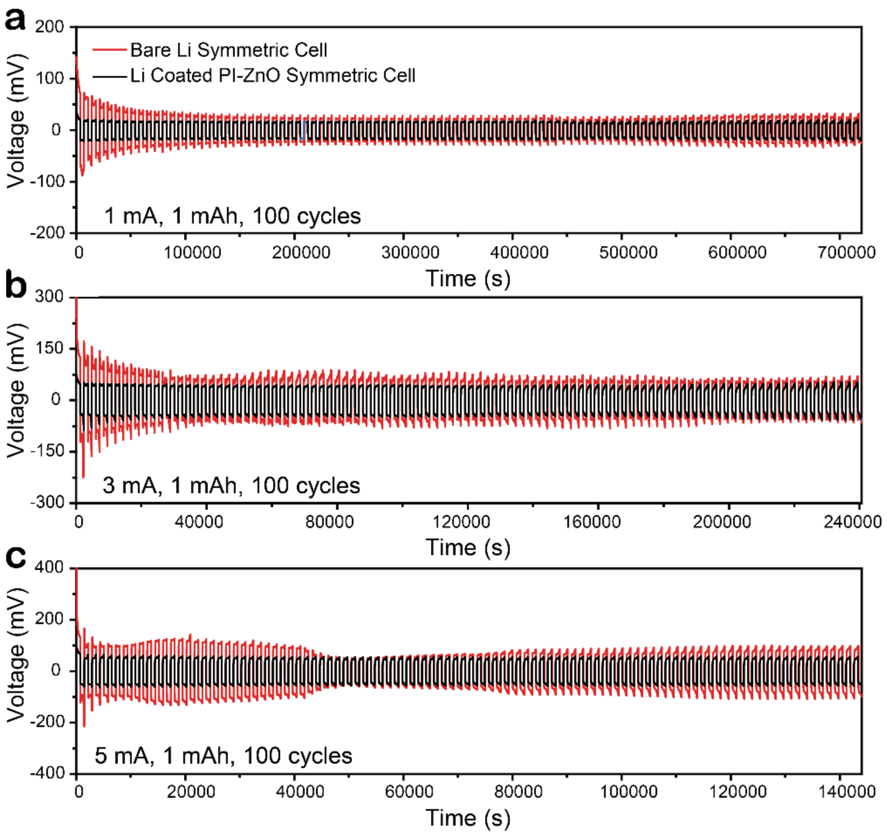

Supplementary Figure 13. Comparison of the cycling stability of the Li-coated PI matrix and the bare Li electrode at a current density of (a) 1 mA cm^{-2}, (b) 3 mA cm^{-2} and (c) 5 mA cm^{-2} in DOL/DME electrolyte. The amount of Li cycled was 1 mAh cm^{-2}. Necking of the voltage profile of the bare Li (overpotential first decreases and then increases) can be observed clearly in (c) due to dendrite growth during cycling, which reduces the effective current density, leading to reduced overpotential.

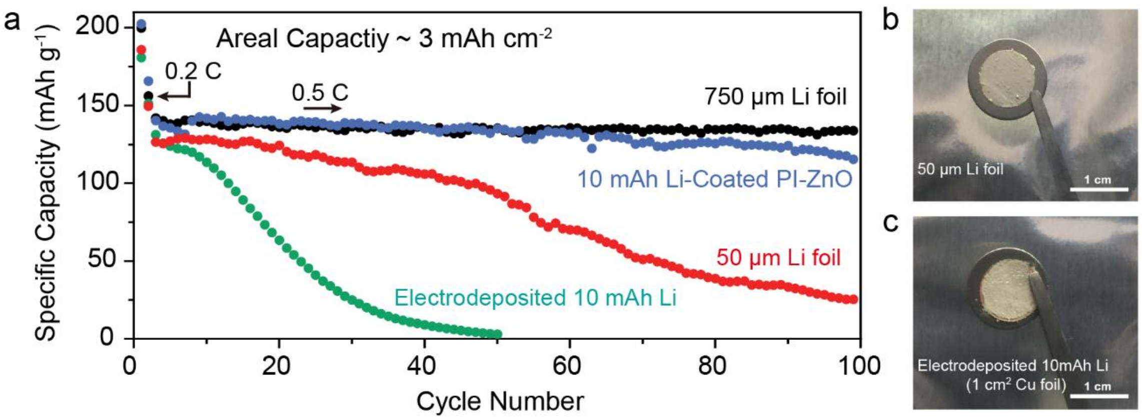

Supplementary Figure 14. (a) Discharge capacity of various Li metal anode-lithium titanate (LTO) cathode full cells for the first 100 galvanostatic cycles in EC/DEC with 1 vol % vinylene carbonate. Rate was set at 0.2 C for the first 2 cycles and 0.5 C for later cycles (1 C = 170 mA g^{-1}). The areal capacity of the cathodes were approximately 3 mAh/cm^2. (b) Photo of the 50 μm Li foil used (capacity ~ 10 mAh cm^{-2}). (c) Photo of the electrodeposited 10 mAh/cm^2 Li used (electrodeposition current density 0.2 mA cm^{-2}).

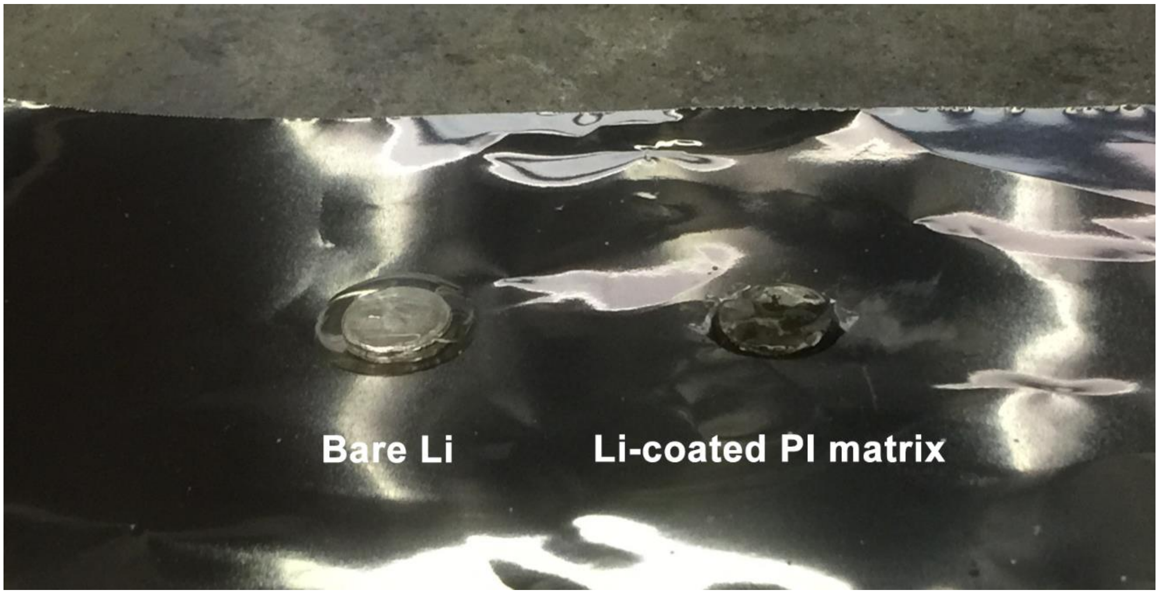

Supplementary Figure 15. Photo of a Li-coated PI matrix and a bare Li electrode with 10 µl electrolyte. The Li-coated PI matrix can uptake a large amount of electrolyte due to its porous nature.

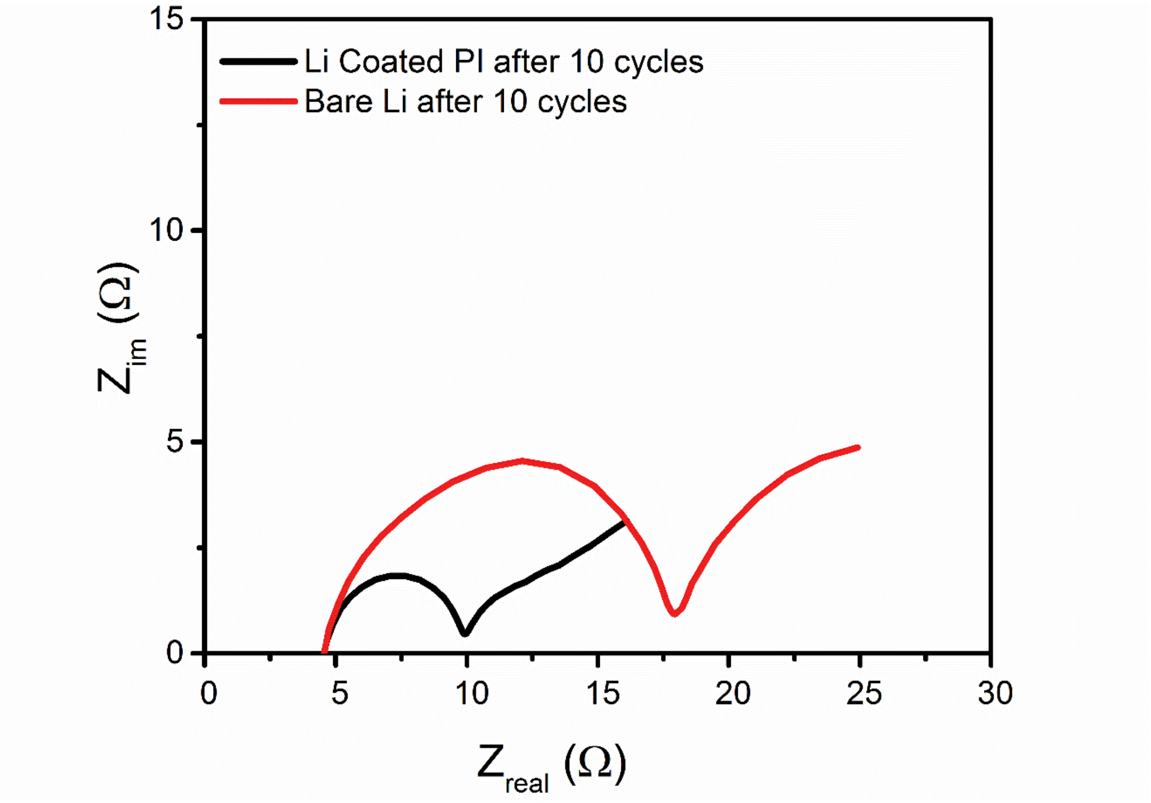

Supplementary Figure 16. Nyquist plot of the impedance spectra of the symmetrical Li-coated PI matrix and the bare Li cell after 10 cycles in DOL/DME at a current density of 1 mA cm^{-2}.

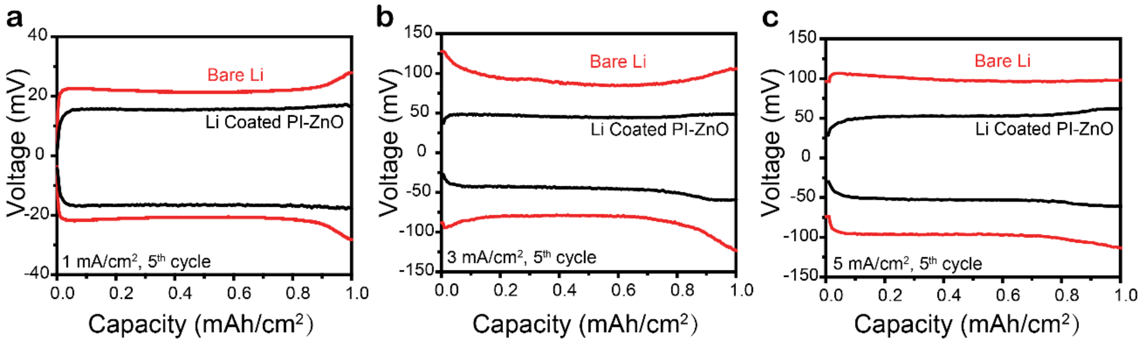

Supplementary Figure 17. Voltage profiles of Li-coated PI matrix and bare Li electrode at a current density of (a) 1 mA cm^{-2}, (b) 3 mA cm^{-2} and (c) 5 mA cm^{-2} after 5 cycles. The amount of Li cycled was 1 mAh cm^{-2} in all cases.

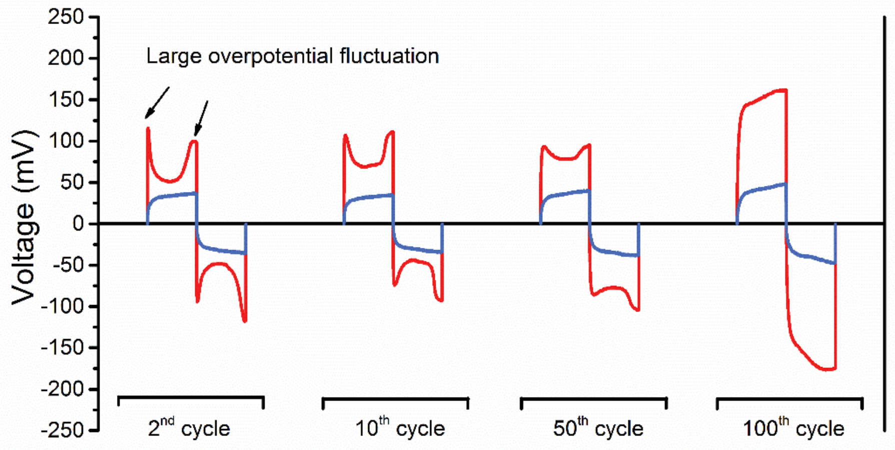

Supplementary Figure 18. Selected overpotential profiles of the Li-coated PI electrode and the bare Li electrode in EC/DEC at a current density of 1 mA cm^{-2}. The Li-coated electrode exhibited a flat stripping/plating profile while that of the bare Li electrode showed large "overpotential bumps". The fluctuation attenuated in later cycles due to the formation of Li dendrites that roughened the surface and reduced the effective current density.

Supplementary Notes

Supplementary Note 1. Semi-quantitative determination of the Coulombic efficiency of electrodes with pre-stored Li.

It is not easy to directly determine the Coulombic efficiency (CE) of electrodes with pre-stored Li through Li plating/stripping as previous studies.[1, 2] To study the CE of our electrode, a counter electrode needed to be carefully selected. We think the counter needs to satisfy two criteria. (i) No Li is pre-stored in the counter. If more Li is stored in the counter, it is challenging to determine the amount of Li is lost during each cycle since extra Li is kept supplying. (ii) The counter electrode itself should always have near 100% CE even at the initial cycles so that all the Li lost can be attributed to the Li metal electrode we are interested in.

Unfortuanately, after searching the potential candidates as the counter, none can perfectly satisfy the above-mentioned criteria. The cloest material we found is $Li_4Ti_5O_{12}$ (LTO), which does not have pre-stored Li inside and has high enough CE in the later cycles. However, it also has two problems, namely, relatively low CE at initial cycles (~90%) and low specific capacity (~170 mAh g^{-1}). Li loss at LTO side in the initial cycles is not negligible and it is challenging preparing high areal capacity electrode of LTO to match high areal capacity of Li metal electrode.

To compromise these problems and semi-quantitatively evaluate the CE of our Li metal electrode, we designed a subtle experiment here (Supplementary Fig. 10). High areal capacity LTO electrode of ~3 mAh cm^{-2} (Commercial level, highest that can be fabricated in our research lab) was used as the counter electrode. Li metal electrode with higher areal capacity of ~ 10 mAh cm^{-2} was paired with the above-mentioned LTO electrode. The cycle life of the cells with this configuration can well reflect the CE of the Li metal electrode. Considering that the LTO electrode is extremely stable without obvious decay in the first 100 cycles, if the Li metal electrode has a CE of ~90%, 0.3 mAh cm^{-2} (10% of 3 mAh cm^{-2}) of Li will be lost at each cycle. As a consequence, the cell will start to decay at ~23 cycles (0.3 mA cm^{-2}*23= extra 7 mAh cm^{-2} of Li). If the CE is lower, the cycle life will be even shorter. (80%-12cycles, 70%-8cycles)

Using the above-mentioned method, we found that our Li-coated PI-ZnO electrode exhibited quite stable cycling. No obvious decay can be observed in the first 65 cycles compared with highly oversized Li metal counterpart (750 μm Li). This illustrates that the CE of our electrode is ~96.4%. In contrast, the bare 50 μm Li foil starts to decay at ~20 cycles, which indicates that the CE is ~88.3%. The electrodeposited 10 mAh Li electrode performed even worse, which started to decay at ~8 cycles, which indicates that its CE is ~70%. The semi-quantitative CE value got on Li foil electrode with this method is similar to that reported before (~76% CE),[2] which proves the validity of our method.

Supplementary Methods

Calculation of the change in electrode dimension during stripping/plating for bare Li.

Theoretical specific capacity of Li is 3860 mAh/g

Density of Li is 0.534 g/cm^3

Volumetric capacity of Li = Theoretical specific capacity of Li $\times$ Density of Li

$$= 2061.24\ mAh/cm^3 \tag{1}$$

Thickness of Li corresponding to 1 mAh/cm^2 capacity = 1 mAh/cm^2 / Volumetric capacity

$$\approx 4.85\ \mu m \tag{2}$$

Supplementary References

1. Zheng, G. Y., *et al.* Interconnected hollow carbon nanospheres for stable lithium metal anodes. *Nat. Nanotech.* **9**, 618-623 (2014).

2. Ding, F., *et al.* Dendrite-free lithium deposition via self-healing electrostatic shield mechanism. *J. Am. Chem. Soc.* **135**, 4450-4456 (2013).
